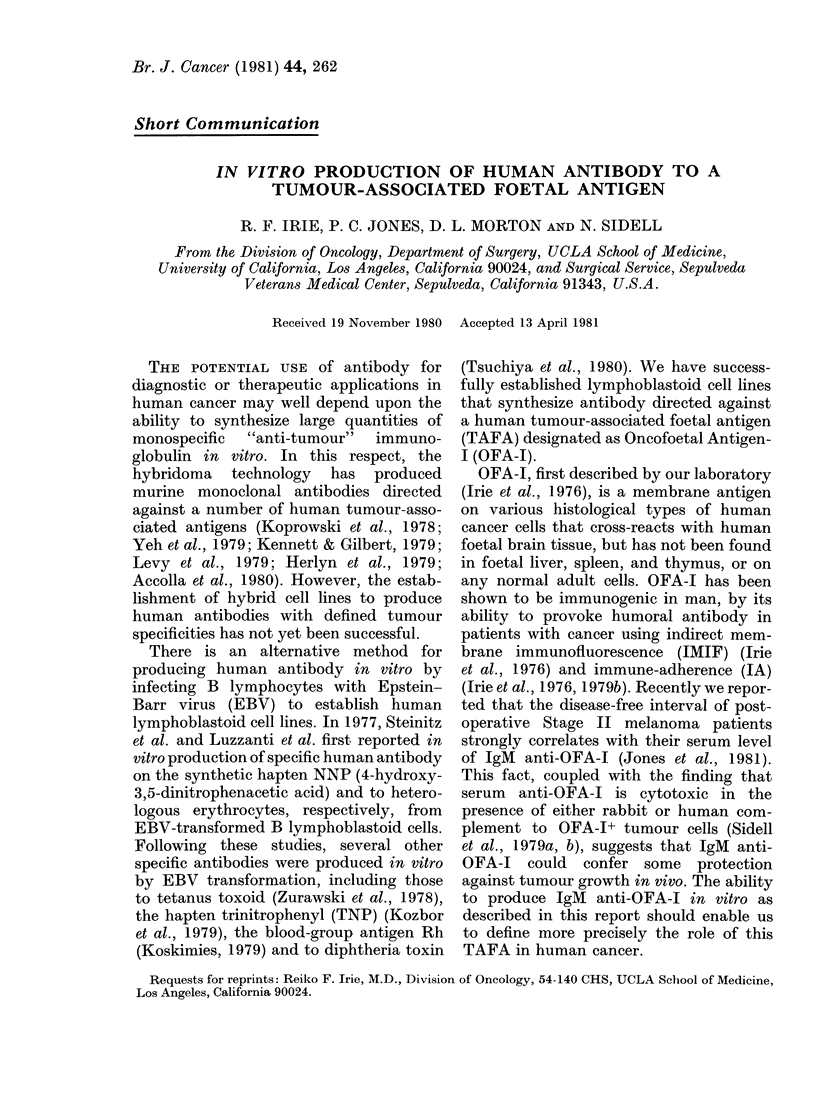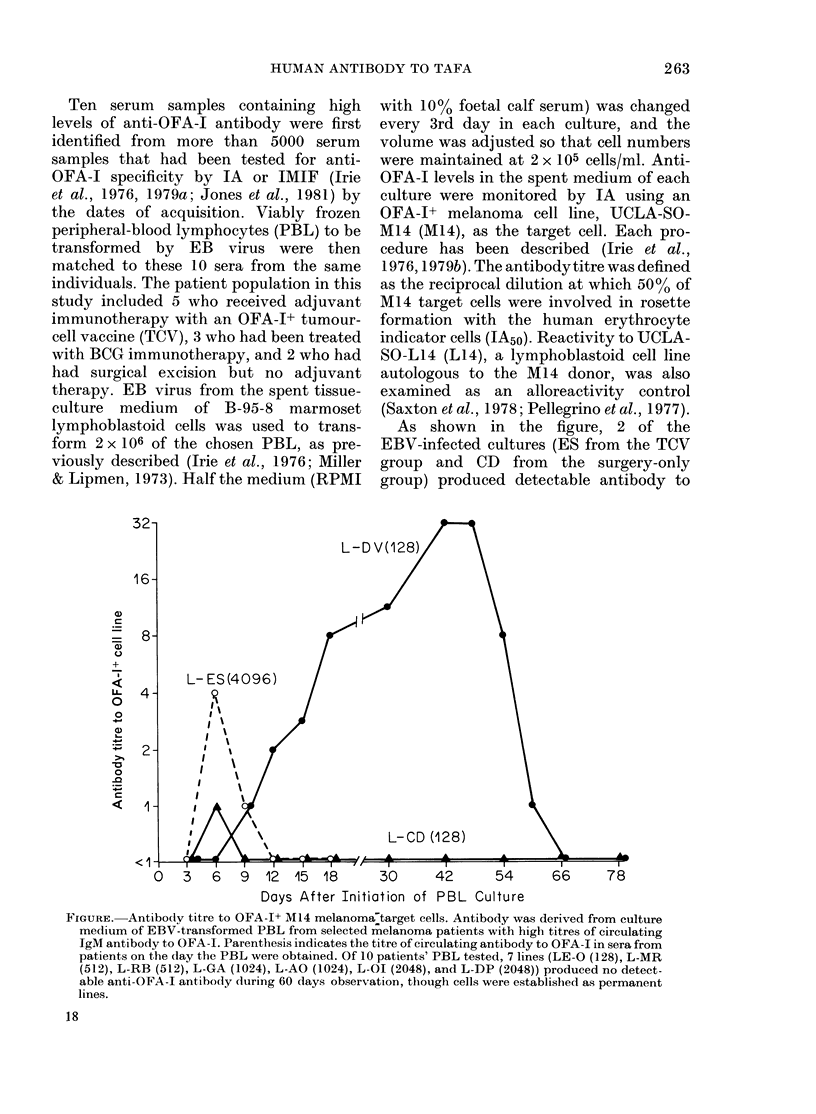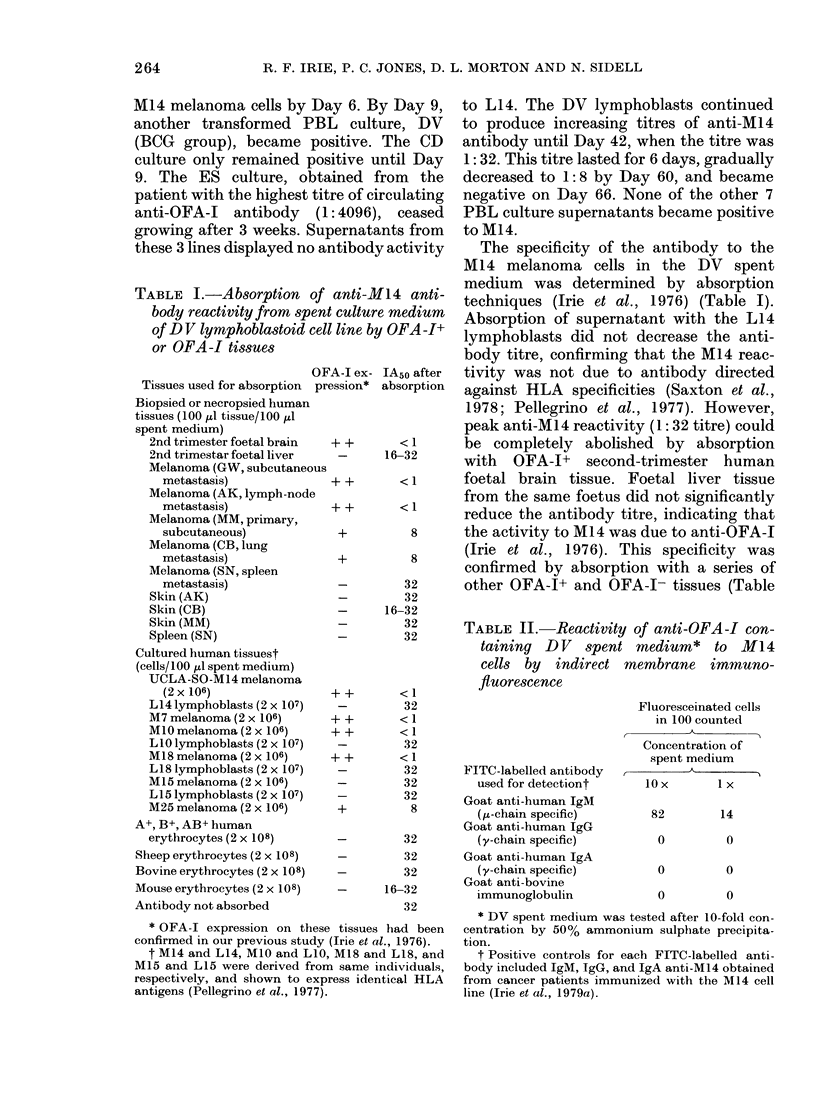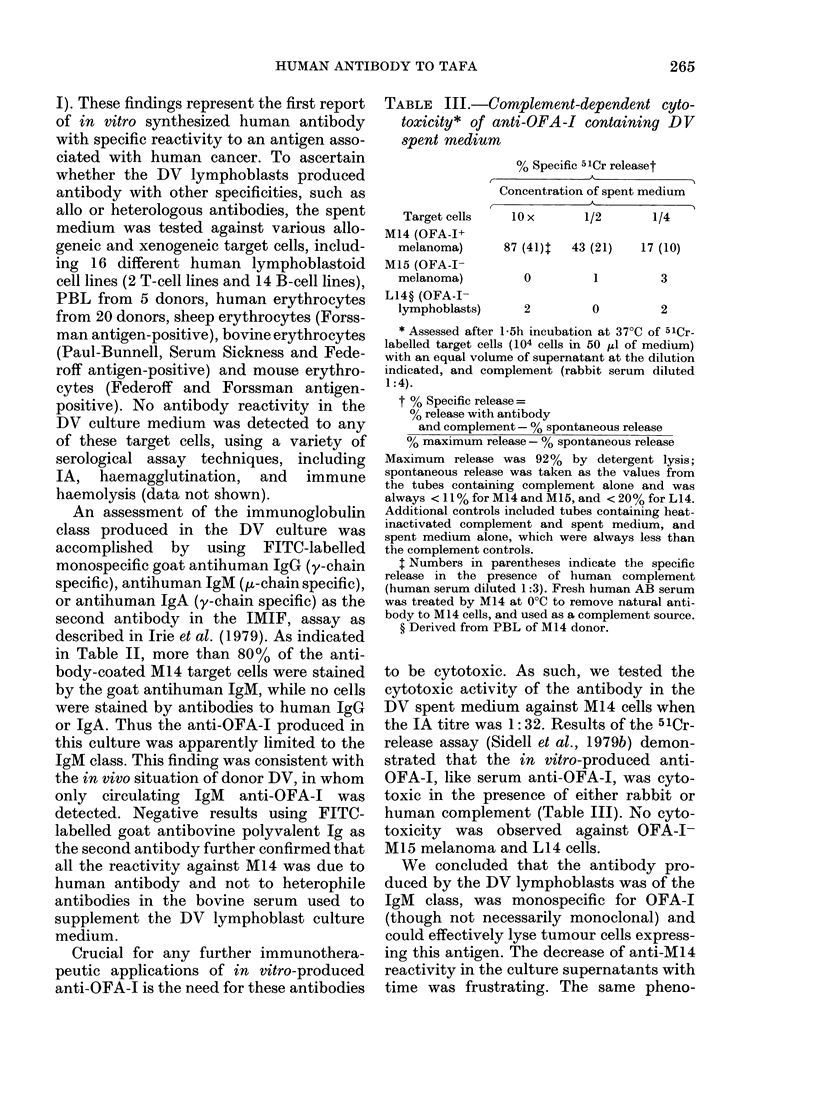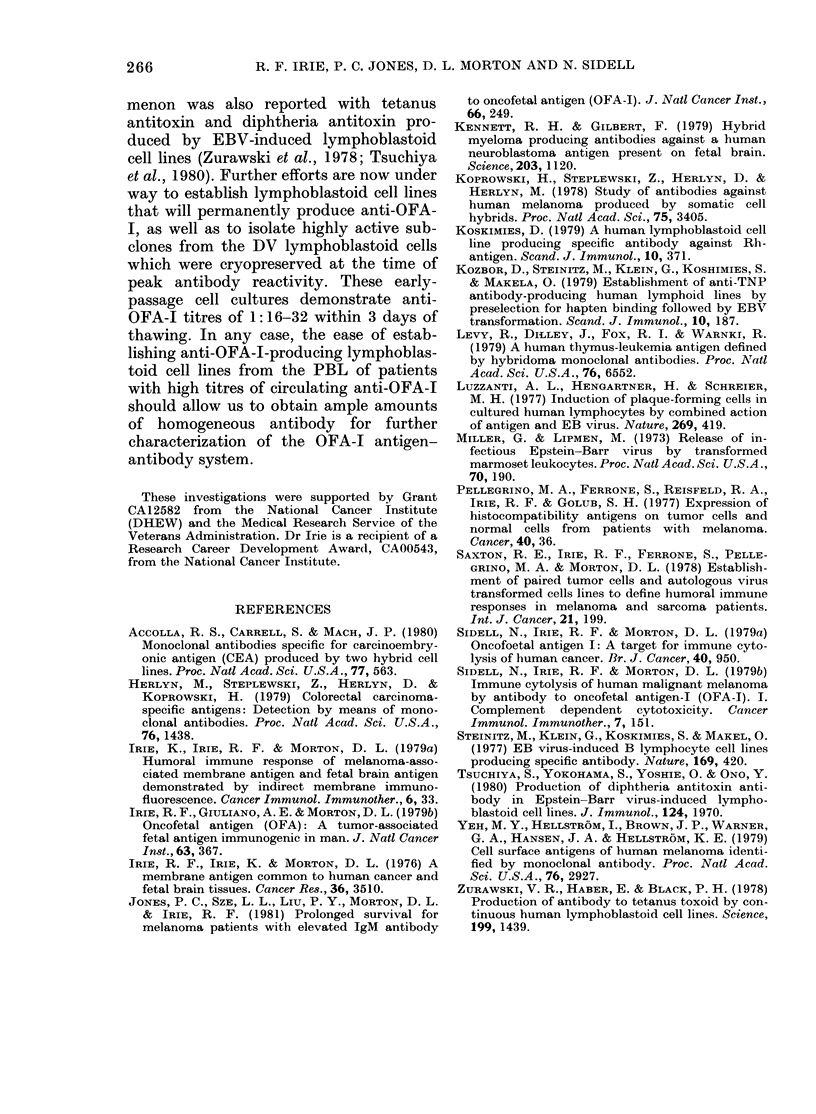# In vitro production of human antibody to a tumour-associated foetal antigen.

**DOI:** 10.1038/bjc.1981.178

**Published:** 1981-08

**Authors:** R. F. Irie, P. C. Jones, D. L. Morton, N. Sidell


*Br. J. Cancer* (1981) **44**, 262

### *Short Communication*

### *IN VITRO* PRODUCTION OF HUMAN ANTIBODY TO A TUMOUR-ASSOCIATED FOETAL ANTIGEN

R. F. IRIE, P. C. JONES, D. L. MORTON AND N. SIDELL

*From the Division of Oncology, Department of Surgery, UCLA School of Medicine, University of California, Los Angeles, California* 90024, *and Surgical Service, Sepulveda Veterans Medical Center, Sepulveda, California* 91343, *U.S.A.*

Received 19 November 1980 Accepted 13 April 1981

THE POTENTIAL USE of antibody for diagnostic or therapeutic applications in human cancer may well depend upon the ability to synthesize large quantities of monospecific "anti-tumour" immunoglobulin *in vitro*. In this respect, the hybridoma technology has produced murine monoclonal antibodies directed against a number of human tumour-associated antigens (Koprowski *et al.*, 1978; Yeh *et al.*, 1979; Kennett & Gilbert, 1979; Levy *et al.*, 1979; Herlyn *et al.*, 1979; Accolla *et al.*, 1980). However, the establishment of hybrid cell lines to produce human antibodies with defined tumour specificities has not yet been successful.

There is an alternative method for producing human antibody *in vitro* by infecting B lymphocytes with Epstein–Barr virus (EBV) to establish human lymphoblastoid cell lines. In 1977, Steinitz *et al.* and Luzzanti *et al.* first reported *in vitro* production of specific human antibody on the synthetic hapten NNP (4-hydroxy-3,5-dinitrophenacetic acid) and to heterologous erythrocytes, respectively, from EBV-transformed B lymphoblastoid cells. Following these studies, several other specific antibodies were produced *in vitro* by EBV transformation, including those to tetanus toxoid (Zurawski *et al.*, 1978), the hapten trinitrophenyl (TNP) (Kozbor *et al.*, 1979), the blood-group antigen Rh (Koskimies, 1979) and to diphtheria toxin (Tsuchiya *et al.*, 1980). We have successfully established lymphoblastoid cell lines that synthesize antibody directed against a human tumour-associated foetal antigen (TAFA) designated as Oncofoetal Antigen-I (OFA-I).

OFA-I, first described by our laboratory (Irie *et al.*, 1976), is a membrane antigen on various histological types of human cancer cells that cross-reacts with human foetal brain tissue, but has not been found in foetal liver, spleen, and thymus, or on any normal adult cells. OFA-I has been shown to be immunogenic in man, by its ability to provoke humoral antibody in patients with cancer using indirect membrane immunofluorescence (IMIF) (Irie *et al.*, 1976) and immune-adherence (IA) (Irie *et al.*, 1976, 1979*b*). Recently we reported that the disease-free interval of post-operative Stage II melanoma patients strongly correlates with their serum level of IgM anti-OFA-I (Jones *et al.*, 1981). This fact, coupled with the finding that serum anti-OFA-I is cytotoxic in the presence of either rabbit or human complement to OFA-I$^{+}$ tumour cells (Sidell *et al.*, 1979*a*, *b*), suggests that IgM anti-OFA-I could confer some protection against tumour growth *in vivo*. The ability to produce IgM anti-OFA-I *in vitro* as described in this report should enable us to define more precisely the role of this TAFA in human cancer.

Requests for reprints: Reiko F. Irie, M.D., Division of Oncology, 54-140 CHS, UCLA School of Medicine, Los Angeles, California 90024.

Ten serum samples containing high levels of anti-OFA-I antibody were first identified from more than 5000 serum samples that had been tested for anti-OFA-I specificity by IA or IMIF (Irie *et al.*, 1976, 1979*a*; Jones *et al.*, 1981) by the dates of acquisition. Viably frozen peripheral-blood lymphocytes (PBL) to be transformed by EB virus were then matched to these 10 sera from the same individuals. The patient population in this study included 5 who received adjuvant immunotherapy with an OFA-I$^+$ tumour-cell vaccine (TCV), 3 who had been treated with BCG immunotherapy, and 2 who had had surgical excision but no adjuvant therapy. EB virus from the spent tissue-culture medium of B-95-8 marmoset lymphoblastoid cells was used to transform $2 \times 10^6$ of the chosen PBL, as previously described (Irie *et al.*, 1976; Miller & Lipmen, 1973). Half the medium (RPMI with 10% foetal calf serum) was changed every 3rd day in each culture, and the volume was adjusted so that cell numbers were maintained at $2 \times 10^5$ cells/ml. Anti-OFA-I levels in the spent medium of each culture were monitored by IA using an OFA-I$^+$ melanoma cell line, UCLA-SO-M14 (M14), as the target cell. Each procedure has been described (Irie *et al.*, 1976, 1979*b*). The antibody titre was defined as the reciprocal dilution at which 50% of M14 target cells were involved in rosette formation with the human erythrocyte indicator cells ($IA_{50}$). Reactivity to UCLA-SO-L14 (L14), a lymphoblastoid cell line autologous to the M14 donor, was also examined as an alloreactivity control (Saxton *et al.*, 1978; Pellegrino *et al.*, 1977).

As shown in the figure, 2 of the EBV-infected cultures (ES from the TCV group and CD from the surgery-only group) produced detectable antibody to

FIGURE.—Antibody titre to OFA-I$^+$ M14 melanoma target cells. Antibody was derived from culture medium of EBV-transformed PBL from selected melanoma patients with high titres of circulating IgM antibody to OFA-I. Parenthesis indicates the titre of circulating antibody to OFA-I in sera from patients on the day the PBL were obtained. Of 10 patients' PBL tested, 7 lines (LE-O (128), L-MR (512), L-RB (512), L-GA (1024), L-AO (1024), L-OI (2048), and L-DP (2048)) produced no detectable anti-OFA-I antibody during 60 days observation, though cells were established as permanent lines.

M14 melanoma cells by Day 6. By Day 9, another transformed PBL culture, DV (BCG group), became positive. The CD culture only remained positive until Day 9. The ES culture, obtained from the patient with the highest titre of circulating anti-OFA-I antibody (1:4096), ceased growing after 3 weeks. Supernatants from these 3 lines displayed no antibody activity to L14. The DV lymphoblasts continued to produce increasing titres of anti-M14 antibody until Day 42, when the titre was 1:32. This titre lasted for 6 days, gradually decreased to 1:8 by Day 60, and became negative on Day 66. None of the other 7 PBL culture supernatants became positive to M14.

The specificity of the antibody to the M14 melanoma cells in the DV spent medium was determined by absorption techniques (Irie *et al.*, 1976) (Table I). Absorption of supernatant with the L14 lymphoblasts did not decrease the antibody titre, confirming that the M14 reactivity was not due to antibody directed against HLA specificities (Saxton *et al.*, 1978; Pellegrino *et al.*, 1977). However, peak anti-M14 reactivity (1:32 titre) could be completely abolished by absorption with $OFA\text{-}I^+$ second-trimester human foetal brain tissue. Foetal liver tissue from the same foetus did not significantly reduce the antibody titre, indicating that the activity to M14 was due to anti-OFA-I (Irie *et al.*, 1976). This specificity was confirmed by absorption with a series of other $OFA\text{-}I^+$ and $OFA\text{-}I^-$ tissues (Table

TABLE I.—*Absorption of anti-M14 antibody reactivity from spent culture medium of DV lymphoblastoid cell line by* $OFA\text{-}I^+$ *or OFA-I tissues*

| Tissues used for absorption | OFA-I expression* | $IA_{50}$ after absorption |
|---|---|---|
| Biopsied or necropsied human tissues (100 μl tissue/100 μl spent medium) | | |
| 2nd trimester foetal brain | + + | < 1 |
| 2nd trimestar foetal liver | − | 16–32 |
| Melanoma (GW, subcutaneous metastasis) | + + | < 1 |
| Melanoma (AK, lymph-node metastasis) | + + | < 1 |
| Melanoma (MM, primary, subcutaneous) | + | 8 |
| Melanoma (CB, lung metastasis) | + | 8 |
| Melanoma (SN, spleen metastasis) | − | 32 |
| Skin (AK) | − | 32 |
| Skin (CB) | − | 16–32 |
| Skin (MM) | − | 32 |
| Spleen (SN) | − | 32 |
| Cultured human tissues† (cells/100 μl spent medium) | | |
| UCLA-SO-M14 melanoma ($2 \times 10^6$) | + + | < 1 |
| L14 lymphoblasts ($2 \times 10^7$) | − | 32 |
| M7 melanoma ($2 \times 10^6$) | + + | < 1 |
| M10 melanoma ($2 \times 10^6$) | + + | < 1 |
| L10 lymphoblasts ($2 \times 10^7$) | − | 32 |
| M18 melanoma ($2 \times 10^6$) | + + | < 1 |
| L18 lymphoblasts ($2 \times 10^7$) | − | 32 |
| M15 melanoma ($2 \times 10^6$) | − | 32 |
| L15 lymphoblasts ($2 \times 10^7$) | − | 32 |
| M25 melanoma ($2 \times 10^6$) | + | 8 |
| $A^+$, $B^+$, $AB^+$ human erythrocytes ($2 \times 10^8$) | − | 32 |
| Sheep erythrocytes ($2 \times 10^8$) | − | 32 |
| Bovine erythrocytes ($2 \times 10^8$) | − | 32 |
| Mouse erythrocytes ($2 \times 10^8$) | − | 16–32 |
| Antibody not absorbed | | 32 |

* OFA-I expression on these tissues had been confirmed in our previous study (Irie *et al.*, 1976).

† M14 and L14, M10 and L10, M18 and L18, and M15 and L15 were derived from same individuals, respectively, and shown to express identical HLA antigens (Pellegrino *et al.*, 1977).

TABLE II.—*Reactivity of anti-OFA-I containing DV spent medium* to M14 cells by indirect membrane immunofluorescence*

| FITC-labelled antibody used for detection† | Fluoresceinated cells in 100 counted — Concentration of spent medium: 10 × | 1 × |
|---|---|---|
| Goat anti-human IgM (μ-chain specific) | 82 | 14 |
| Goat anti-human IgG (γ-chain specific) | 0 | 0 |
| Goat anti-human IgA (γ-chain specific) | 0 | 0 |
| Goat anti-bovine immunoglobulin | 0 | 0 |

* DV spent medium was tested after 10-fold concentration by 50% ammonium sulphate precipitation.

† Positive controls for each FITC-labelled antibody included IgM, IgG, and IgA anti-M14 obtained from cancer patients immunized with the M14 cell line (Irie *et al.*, 1979*a*).

I). These findings represent the first report of *in vitro* synthesized human antibody with specific reactivity to an antigen associated with human cancer. To ascertain whether the DV lymphoblasts produced antibody with other specificities, such as allo or heterologous antibodies, the spent medium was tested against various allogeneic and xenogeneic target cells, including 16 different human lymphoblastoid cell lines (2 T-cell lines and 14 B-cell lines), PBL from 5 donors, human erythrocytes from 20 donors, sheep erythrocytes (Forssman antigen-positive), bovine erythrocytes (Paul-Bunnell, Serum Sickness and Federoff antigen-positive) and mouse erythrocytes (Federoff and Forssman antigen-positive). No antibody reactivity in the DV culture medium was detected to any of these target cells, using a variety of serological assay techniques, including IA, haemagglutination, and immune haemolysis (data not shown).

An assessment of the immunoglobulin class produced in the DV culture was accomplished by using FITC-labelled monospecific goat antihuman IgG ($\gamma$-chain specific), antihuman IgM ($\mu$-chain specific), or antihuman IgA ($\gamma$-chain specific) as the second antibody in the IMIF, assay as described in Irie *et al.* (1979). As indicated in Table II, more than 80% of the antibody-coated M14 target cells were stained by the goat antihuman IgM, while no cells were stained by antibodies to human IgG or IgA. Thus the anti-OFA-I produced in this culture was apparently limited to the IgM class. This finding was consistent with the *in vivo* situation of donor DV, in whom only circulating IgM anti-OFA-I was detected. Negative results using FITC-labelled goat antibovine polyvalent Ig as the second antibody further confirmed that all the reactivity against M14 was due to human antibody and not to heterophile antibodies in the bovine serum used to supplement the DV lymphoblast culture medium.

Crucial for any further immunotherapeutic applications of *in vitro*-produced anti-OFA-I is the need for these antibodies to be cytotoxic. As such, we tested the cytotoxic activity of the antibody in the DV spent medium against M14 cells when the IA titre was 1:32. Results of the $^{51}$Cr-release assay (Sidell *et al.*, 1979*b*) demonstrated that the *in vitro*-produced anti-OFA-I, like serum anti-OFA-I, was cytotoxic in the presence of either rabbit or human complement (Table III). No cytotoxicity was observed against OFA-I$^-$ M15 melanoma and L14 cells.

We concluded that the antibody produced by the DV lymphoblasts was of the IgM class, was monospecific for OFA-I (though not necessarily monoclonal) and could effectively lyse tumour cells expressing this antigen. The decrease of anti-M14 reactivity in the culture supernatants with time was frustrating. The same pheno-

TABLE III.—*Complement-dependent cytotoxicity* of anti-OFA-I containing DV spent medium*

| | % Specific $^{51}$Cr release† | | |
|---|---|---|---|
| | Concentration of spent medium | | |
| Target cells | 10× | 1/2 | 1/4 |
| M14 (OFA-I$^+$ melanoma) | 87 (41)‡ | 43 (21) | 17 (10) |
| M15 (OFA-I$^-$ melanoma) | 0 | 1 | 3 |
| L14§ (OFA-I$^-$ lymphoblasts) | 2 | 0 | 2 |

* Assessed after 1·5h incubation at 37°C of $^{51}$Cr-labelled target cells ($10^4$ cells in 50 $\mu$l of medium) with an equal volume of supernatant at the dilution indicated, and complement (rabbit serum diluted 1:4).

† % Specific release =

$$\frac{\text{\% release with antibody and complement} - \text{\% spontaneous release}}{\text{\% maximum release} - \text{\% spontaneous release}}$$

Maximum release was 92% by detergent lysis; spontaneous release was taken as the values from the tubes containing complement alone and was always < 11% for M14 and M15, and < 20% for L14. Additional controls included tubes containing heat-inactivated complement and spent medium, and spent medium alone, which were always less than the complement controls.

‡ Numbers in parentheses indicate the specific release in the presence of human complement (human serum diluted 1:3). Fresh human AB serum was treated by M14 at 0°C to remove natural antibody to M14 cells, and used as a complement source.

§ Derived from PBL of M14 donor.

menon was also reported with tetanus antitoxin and diphtheria antitoxin produced by EBV-induced lymphoblastoid cell lines (Zurawski *et al.*, 1978; Tsuchiya *et al.*, 1980). Further efforts are now under way to establish lymphoblastoid cell lines that will permanently produce anti-OFA-I, as well as to isolate highly active subclones from the DV lymphoblastoid cells which were cryopreserved at the time of peak antibody reactivity. These early-passage cell cultures demonstrate anti-OFA-I titres of 1:16–32 within 3 days of thawing. In any case, the ease of establishing anti-OFA-I-producing lymphoblastoid cell lines from the PBL of patients with high titres of circulating anti-OFA-I should allow us to obtain ample amounts of homogeneous antibody for further characterization of the OFA-I antigen–antibody system.

These investigations were supported by Grant CA12582 from the National Cancer Institute (DHEW) and the Medical Research Service of the Veterans Administration. Dr Irie is a recipient of a Research Career Development Award, CA00543, from the National Cancer Institute.

### REFERENCES

Accolla, R. S., Carrell, S. & Mach, J. P. (1980) Monoclonal antibodies specific for carcinoembryonic antigen (CEA) produced by two hybrid cell lines. *Proc. Natl Acad. Sci. U.S.A.*, **77**, 563.

Herlyn, M., Steplewski, Z., Herlyn, D. & Koprowski, H. (1979) Colorectal carcinoma-specific antigens: Detection by means of monoclonal antibodies. *Proc. Natl Acad. Sci. U.S.A.*, **76**, 1438.

Irie, K., Irie, R. F. & Morton, D. L. (1979*a*) Humoral immune response of melanoma-associated membrane antigen and fetal brain antigen demonstrated by indirect membrane immunofluorescence. *Cancer Immunol. Immunother.*, **6**, 33.

Irie, R. F., Giuliano, A. E. & Morton, D. L. (1979*b*) Oncofetal antigen (OFA): A tumor-associated fetal antigen immunogenic in man. *J. Natl Cancer Inst.*, **63**, 367.

Irie, R. F., Irie, K. & Morton, D. L. (1976) A membrane antigen common to human cancer and fetal brain tissues. *Cancer Res.*, **36**, 3510.

Jones, P. C., Sze, L. L., Liu, P. Y., Morton, D. L. & Irie, R. F. (1981) Prolonged survival for melanoma patients with elevated IgM antibody to oncofetal antigen (OFA-I). *J. Natl Cancer Inst.*, **66**, 249.

Kennett, R. H. & Gilbert, F. (1979) Hybrid myeloma producing antibodies against a human neuroblastoma antigen present on fetal brain. *Science*, **203**, 1120.

Koprowski, H., Steplewski, Z., Herlyn, D. & Herlyn, M. (1978) Study of antibodies against human melanoma produced by somatic cell hybrids. *Proc. Natl Acad. Sci.*, **75**, 3405.

Koskimies, D. (1979) A human lymphoblastoid cell line producing specific antibody against Rh-antigen. *Scand. J. Immunol.*, **10**, 371.

Kozbor, D., Steinitz, M., Klein, G., Koshimies, S. & Makela, O. (1979) Establishment of anti-TNP antibody-producing human lymphoid lines by preselection for hapten binding followed by EBV transformation. *Scand. J. Immunol.*, **10**, 187.

Levy, R., Dilley, J., Fox, R. I. & Warnki, R. (1979) A human thymus-leukemia antigen defined by hybridoma monoclonal antibodies. *Proc. Natl Acad. Sci. U.S.A.*, **76**, 6552.

Luzzanti, A. L., Hengartner, H. & Schreier, M. H. (1977) Induction of plaque-forming cells in cultured human lymphocytes by combined action of antigen and EB virus. *Nature*, **269**, 419.

Miller, G. & Lipmen, M. (1973) Release of infectious Epstein–Barr virus by transformed marmoset leukocytes. *Proc. Natl Acad. Sci. U.S.A.*, **70**, 190.

Pellegrino, M. A., Ferrone, S., Reisfeld, R. A., Irie, R. F. & Golub, S. H. (1977) Expression of histocompatibility antigens on tumor cells and normal cells from patients with melanoma. *Cancer*, **40**, 36.

Saxton, R. E., Irie, R. F., Ferrone, S., Pellegrino, M. A. & Morton, D. L. (1978) Establishment of paired tumor cells and autologous virus transformed cells lines to define humoral immune responses in melanoma and sarcoma patients. *Int. J. Cancer*, **21**, 199.

Sidell, N., Irie, R. F. & Morton, D. L. (1979*a*) Oncofoetal antigen I: A target for immune cytolysis of human cancer. *Br. J. Cancer*, **40**, 950.

Sidell, N., Irie, R. F. & Morton, D. L. (1979*b*) Immune cytolysis of human malignant melanoma by antibody to oncofetal antigen-I (OFA-I). I. Complement dependent cytotoxicity. *Cancer Immunol. Immunother.*, **7**, 151.

Steinitz, M., Klein, G., Koskimies, S. & Makel, O. (1977) EB virus-induced B lymphocyte cell lines producing specific antibody. *Nature*, **169**, 420.

Tsuchiya, S., Yokohama, S., Yoshie, O. & Ono, Y. (1980) Production of diphtheria antitoxin antibody in Epstein–Barr virus-induced lymphoblastoid cell lines. *J. Immunol.*, **124**, 1970.

Yeh, M. Y., Hellström, I., Brown, J. P., Warner, G. A., Hansen, J. A. & Hellström, K. E. (1979) Cell surface antigens of human melanoma identified by monoclonal antibody. *Proc. Natl Acad. Sci. U.S.A.*, **76**, 2927.

Zurawski, V. R., Haber, E. & Black, P. H. (1978) Production of antibody to tetanus toxoid by continuous human lymphoblastoid cell lines. *Science*, **199**, 1439.